# IVF or ICSI for fertility preservation?

**DOI:** 10.1530/RAF-20-0059

**Published:** 2021-03-05

**Authors:** Bhorika Aggarwal, Amanda L Evans, Howard Ryan, Sarah J Martins da Silva

**Affiliations:** 1 Reproductive Medicine Research Group, School of Medicine, Ninewells Hospital and Medical School, University of Dundee, Dundee, UK; 2 Human Fertilisation and Embryology Authority (HFEA), London, UK

## Abstract

In IVF, eggs and sperm are added together for fertilisation to occur whereas ICSI involves injecting a single sperm into each egg. ICSI is very effective where sperm count or swimming is poor (male infertility) but is slightly riskier than IVF in terms of health problems in children, although these risks are small. However, the risk of no eggs fertilising is higher for IVF compared to ICSI and couples undertaking fertility preservation, for example, before cancer treatment, usually only have time for one attempt. Using fertility preservation treatment cycle data reported to Human Fertilisation and Embryology Authority (HFEA), this study shows that ICSI results in higher number of fertilised eggs and embryos for storage or treatment compared to IVF. However, 19% of eggs are not used in ICSI treatment, so IVF appears to be better overall. Clinics should choose IVF or ICSI for fertility preservation depending on sperm characteristics rather than using ICSI for all.

Intracytoplasmic sperm injection (ICSI) is highly effective for male factor infertility. However, its use for non-male factor infertility has increased dramatically worldwide in the last 2 decades despite little evidence demonstrating effectiveness in this population. The rationale for using ICSI is to reduce the risk of low or total failed fertilisation (TFF), thereby increasing the number of embryos and the potential for pregnancy and live birth (Bhattacharya *et al.* 2013). A meta-analysis (Johnson *et al.* 2013) of sibling oocyte studies reported a significantly higher pooled relative risk of TFF with IVF compared to ICSI. In contrast, more recent studies of infertile couples with non-male factor infertility show no difference in fertilisation, implantation or pregnancy rates (Li *et al.* 2018), even in poor responder patients (Sfontouris *et al.* 2015) or advanced maternal age (Tannus *et al.* 2017).

Arguing against an approach of ICSI for all there is accumulating information on the health of offspring including, amongst others, increased risk of congenital malformations, chromosomal abnormalities and epigenetic syndromes compared to naturally conceived children (Davies *et al.* 2017, Xiong *et al.* 2017, Esteves *et al.* 2018) and lower sperm concentration in male offspring (Belva *et al.* 2019).

Overall, TFF has been reported to complicate 1–3% ICSI and 5–8% IVF cycles (Swain & Pool 2008). This is particularly relevant for couples undertaking emergency fertility preservation who may only have one opportunity to create embryos. As such, there is a genuine debate regarding the correct approach to fertilisation for this particular group of patients: whether to apply IVF or ICSI depending on sperm characteristics or to undertake ICSI for all. In an attempt to resolve this dilemma, we analysed data provided by Human Fertilisation and Embryo Authority (HFEA). We present data for UK fertility preservation cycles 2015–2018 and 218,830 oocytes retrieved (Table 1), with known insemination method, fertilisation and downstream embryo disposal (transferred, stored, donated). Fertilisation rate (FR) was calculated from the number of oocytes normally fertilised (2PN) divided by the number of inseminated oocytes (IVF) or the number of oocytes microinjected (ICSI).

**Table 1:** Data for all UK fertility preservation cycles reported to HFEA 2015–2018. IVF and ICSI cycles are shown by intention to treat (ITT). Despite ITT, some IVF cycles reported oocyte injection. These were excluded from analysis as numbers were very small and fertilisation data were low (11/33; 33.3% (shown in italics)), raising the possibility of rescue ICSI. Similarly, some ICSI cycles reported conventional oocyte insemination. These were also excluded from further analysis as no further information was available, fertilisation was unexpectedly low (938/5660; 16.6% (shown in italics)). 52.3% normally fertilised eggs (2PN) resulted in embryos for storage, treatment or donation. This was identical whether derived from IVF or ICSI. Overall, 99.2% of embryos were cryostored.

	IVF					ICSI				
	2015	2016	2017	2018	Total	2015	2016	2017	2018	Total
Eggs collected	13,027	17,138	21,825	25,271	77,261	26,316	34,254	37,409	43,590	141,569
Eggs inseminated (IVF)	12,618	16,792	21,277	24,663	75,350	*1368*	*1238*	*1518*	*1536*	*5660*
M2 eggs injected (ICSI)	*14*	*0*	*19*	*0*	*33*	20,313	26,368	28,751	33,469	108,901
Eggs not used	409	346	529	608	1892	4635	6648	7140	8585	27,008
2PN (IVF)	8300	10,756	13,979	15,858	48,893					
2PN (ICSI)						14,643	19,308	21,194	24,126	79,271
Fertilisation rate (%)	65.8	64.1	65.7	64.3	64.9	72.1	73.2	73.7	72.1	72.8
Embryos stored	4917	5752	7331	7437	25,437	8419	10,459	11,045	11,131	41,054
Total embryos: treatment and storage	4934	5782	7373	7501	25,590	8464	10,538	11,177	11,271	41,450
% embryos stored	99.7	99.5	99.4	99.1	99.4	99.5	99.3	98.8	98.8	99.0
% embryos generated from 2PN	59.4	53.8	52.7	47.3	52.3	57.8	54.6	52.7	46.7	52.3

In total, 75,350 eggs were inseminated (IVF) and 108,901 eggs were injected (ICSI). FR was significantly higher for ICSI compared to IVF (72.8% vs 64.9%; *P*  < 0.00001). A significantly higher proportion of embryos resulted from ICSI per egg injected compared to IVF per egg inseminated (38.1% vs 34.0%; *P * < 0.00001). However, 19.1% (27,008) eggs allocated to ICSI were not used, presumably due to immaturity or being otherwise unsuitable for injection, compared to only 2.4% (1878) eggs not used for IVF insemination. The percentage of embryos generated for treatment or storage from normally fertilised eggs (2PN) was identical between IVF and ICSI. Over 99% of all embryos were cryostored.

These data demonstrate that although a 7.9% higher FR is seen with ICSI compared to IVF, this does not compensate for the significantly higher proportion of eggs not used for microinjection, and we ,therefore recommend a strategy of IVF or ICSI depending on sperm characteristics rather than ICSI for all fertility preservation.

## Declaration of interest

Sarah J Martins da Silva is an Associate Editor of Reproduction and Fertility. Sarah J Martins da Silva was not involved in the review or editorial process for this paper on which she is listed as an author.

## Author contribution statement

B A analysed the data. A L E and H R collected and collated HFEA data. S M D S conceived the study. S M D S, B A and A L E wrote the paper.

## References

[bib1] Belva F Bonduelle M Tournaye H 2019 Endocrine and reproductive profile of boys and young adults conceived after ICSI. Current Opinion in Obstetrics and Gynecology 31 163–169. (10.1097/GCO.0000000000000538) 30870183

[bib2] Bhattacharya S Maheshwari A Mollison J 2013 Factors associated with failed treatment: an analysis of 121,744 women embarking on their first IVF cycles. PLoS ONE 8 e82249. (10.1371/journal.pone.0082249) PMC3857793 24349236

[bib3] Davies MJ Rumbold AR Marino JL Willson K Giles LC Whitrow MJ Scheil W Moran LJ Thompson JG Lane M 2017 Maternal factors and the risk of birth defects after IVF and ICSI: a whole of population cohort study. BJOG 124 1537–1544. (10.1111/1471-0528.14365) 27748040

[bib4] Esteves SC Roque M Bedoschi G Haahr T Humaidan P 2018 Intracytoplasmic sperm injection for male infertility and consequences for offspring. Nature Reviews: Urology 15 535–562. (10.1038/s41585-018-0051-8) 29967387

[bib5] Johnson LN Sasson IE Sammel MD Dokras A 2013 Does intracytoplasmic sperm injection improve the fertilization rate and decrease the total fertilization failure rate in couples with well-defined unexplained infertility? A systematic review and meta-analysis. Fertility and Sterility 100 704–711. (10.1016/j.fertnstert.2013.04.038) 23773312

[bib6] Li Z Wang AY Bowman M Hammarberg K Farquhar C Johnson L Safi N Sullivan EA 2018 ICSI does not increase the cumulative live birth rate in non-male factor infertility. Human Reproduction 33 1322–1330. (10.1093/humrep/dey118) 29897449

[bib7] Sfontouris IA Kolibianakis EM Lainas GT Navaratnarajah R Tarlatzis BC Lainas TG 2015 Live birth rates using conventional in vitro fertilization compared to intracytoplasmic sperm injection in Bologna poor responders with a single oocyte retrieved. Journal of Assisted Reproduction and Genetics 32 691–697. (10.1007/s10815-015-0459-5) 25758990 PMC4429441

[bib8] Swain JE Pool TB 2008 ART failure: oocyte contributions to unsuccessful fertilization. Human Reproduction Update 14 431–446. (10.1093/humupd/dmn025) 18603645

[bib9] Tannus S Son WY Gilman A Younes G Shavit T Dahan MH 2017 The role of intracytoplasmic sperm injection in non-male factor infertility in advanced maternal age. Human Reproduction 32 119–124. (10.1093/humrep/dew298) 27852688

[bib10] Xiong X Dickey RP Buekens P Shaffer JG Pridjian G 2017 Use of intracytoplasmic sperm injection and birth outcomes in women conceiving through in vitro fertilization. Paediatric and Perinatal Epidemiology 31 108–115. (10.1111/ppe.12339) 28140471

